# Why Pre-Registration of Research Must Be Taken More Seriously

**DOI:** 10.3390/nursrep13020060

**Published:** 2023-04-12

**Authors:** Richard Gray, Daniel Bressington, David R. Thompson, Martin Jones

**Affiliations:** 1 School of Nursing and Midwifery, La Trobe University, Melbourne 3086, Australia; 2 School of Nursing and Midwifery, Queen’s University Belfast, Belfast BT9 7BL, UK; 3 Department of Rural Health, University of South Australia, Adelaide 5000, Australia

The scientific method assumes that researchers use evidence generated from observational research to make predictions (hypotheses) that can be tested experimentally. In clinical disciplines, this is generally within the context of a randomised controlled trial. Sometimes predictions are correct, in which case replication of the study—ideally by a separate and independent research group—is required. If the prediction is replicated (ideally more than once), a case can be made for changing practice. Where the hypothesis is rejected, researchers may need to go back to their observational work to refine their theory/model and generate new predictions. The method is sound. It works. It is how clinical researchers prove that new treatment X is safe and effective. The method requires that researchers do not become tempted to “fiddle” their predictions—but they do [1]. Even in nursing, ranked the most ethical of professions 21 years in a row [2], there are numerous case examples of outcome manipulation. For example, Kao et al. (2018) [3] reported a randomised controlled trial that aimed to test the effectiveness of interactive cognitive motor training on gait and balance in 62 older adults. The authors concluded that the intervention was effective. However, improvement in gait and balance was not the prediction made by the authors. Originally the authors predicted that the intervention would improve cognitive function. These data were omitted from their reporting, presumably because there was no apparent effect on cognitive functioning (these data are actually reported in a separate paper [4]).

What would motivate a research group to misrepresent the findings of their research? The answer is quite straightforward. Research where the prediction is correct is significantly more likely to be published in a “prestigious” journal than research where the prediction is wrong. The Kao et al. (2018) [3] trial was, indeed, published in the *International Journal of Nursing Studies,* the leading journal in the discipline. Studies where a researcher predicts correctly are also more likely to garner more media attention and ultimately advance the careers of those involved. It is easy to see why, when they realised that their prediction was wrong, Kao et al. (2018) [3] succumbed to temptation and substituted cognition for gait. This is sometimes referred to as the I-knew-it-all-along effect, or hindsight bias, as in “we really knew that it was gait that was the outcome that would change and not cognition” [5]. At the end of the day, does it really matter if there is some minor “tinkering” with the order of outcomes at the end of a study?

Changing predictions does matter, essentially for “statistical reasons”—two words likely to stop you reading and jump to the conclusion of this Editorial. Please don’t. Null hypothesis significance testing—extensively used in clinical research—is based on the assumption that researchers have made a prediction that is being tested, a comparison of a null hypothesis where there is no relationship between variables and a hypothesis where there is [5]. If the research group can reject the null hypothesis (*p* < 0.05), it may be stated that it is plausible that the hypothesis is true. The more statistical tests that are performed, because, for example, there are more outcomes that have been measured, the more likely it is that there will be a *p* value that crosses the (magical, not magical) 0.05 threshold. There are also multiple different ways in which statistical tests can be undertaken that may also be more likely to generate the required *p* value. Changing predictions after the event and analysing data in multiple different ways matters because researchers will argue—with a substantially higher degree of confidence than is warranted—that an intervention is effective. Kao et al. (2018) [3] carry exactly this out in their trial, concluding that their intervention should be applied in clinical practice when, clearly, it should not.

The mechanism for ensuring that researchers stick with their predictions is well understood—pre-registration of research outcome and analysis plan with a registry, such as ANZCTN, clinicaltrials.gov, or the Open Science Framework. By pre-registering their work, researchers place their bets (predictions) with an independent, publicly accessible registry. This means that if there are any deviations between what was planned and what was reported, they can be spotted. The key word here is “can”. The problem is that registries are places where researchers lodge information about their predictions. There is no mechanism to determine if the registration entry and publication match. The burden for this work falls to the journal editor and peer reviewer. It is safe to say that: 1. journal editors and reviewers are already burdened and 2. neither group is aware of the need to check [6]. Consequently, even if a study is pre-registered, the chances of authors being caught if they flip the outcomes are remote. Where authors are caught and have been challenged—as was the case in the Kao et al. (2018) [3] example—the authors can easily deflect against the criticism (see the author explanation of why outcomes were switched [7]).

In clinical disciplines such as nursing, the research we undertake directly impacts patient care. Consequently, it is vitally important that researchers strictly adhere to the scientific method. We consider that researchers, reviewers, and journal editors need to take pre-registration far more seriously than is currently the case. To this end, *Nursing Reports* is mandating that authors include a statement about the registration status of their research when they submit their manuscripts for consideration for publication. Studies not properly registered will be required to include a statement in the limitations section of the manuscript indicating such and advising that readers may consequently infer a high risk of bias.
